# A qualitative study of health system barriers to accessibility and utilization of maternal and newborn healthcare services in Ghana after user-fee abolition

**DOI:** 10.1186/s12884-014-0425-8

**Published:** 2014-12-21

**Authors:** John Kuumuori Ganle, Michael Parker, Raymond Fitzpatrick, Easmon Otupiri

**Affiliations:** Department of Geography and Rural Development, Population, Health and Gender Studies Group, Faculty of Social Sciences, Kwame Nkrumah University of Science and Technology, Kumasi, Ghana; Nuffield Department of Population Health, The Ethox Centre, University of Oxford, Rosemary Rue Building, Old Road Campus, Headington Oxford, OX3 7LF United Kingdom; Nuffield Department of Population Health, University of Oxford, Rosemary Rue Building, Old Road Campus, Headington Oxford, OX3 7LF United Kingdom; Department of Community Health, School of Medical Sciences, Kwame Nkrumah University of Science and Technology, Kumasi, Ghana

**Keywords:** Maternal and newborn health, Health system barriers, Access, Maternal healthcare, User-fee abolition, Ghana

## Abstract

**Background:**

To reduce financial barriers to access, and improve access to and use of skilled maternal and newborn healthcare services, the government of Ghana, in 2003, implemented a new maternal healthcare policy that provided free maternity care services in all public and mission healthcare facilities. Although supervised delivery in Ghana has increased from 47% in 2003 to 55% in 2010, strikingly high maternal mortality ratio and low percentage of skilled attendance are still recorded in many parts of the country.

To explore health system factors that inhibit women’s access to and use of skilled maternal and newborn healthcare services in Ghana despite these services being provided free.

**Methods:**

We conducted qualitative research with 185 expectant and lactating mothers and 20 healthcare providers in six communities in Ghana between November 2011 and May 2012. We used Attride-Stirling’s thematic network analysis framework to analyze and present our data.

**Results:**

We found that in addition to limited and unequal distribution of skilled maternity care services, women’s experiences of intimidation in healthcare facilities, unfriendly healthcare providers, cultural insensitivity, long waiting time before care is received, limited birthing choices, poor care quality, lack of privacy at healthcare facilities, and difficulties relating to arranging suitable transportation were important health system barriers to increased and equitable access and use of services in Ghana.

**Conclusion:**

Our findings highlight how a focus on patient-side factors can conceal the fact that many health systems and maternity healthcare facilities in low-income settings such as Ghana are still chronically under-resourced and incapable of effectively providing an acceptable minimum quality of care in the event of serious obstetric complications. Efforts to encourage continued use of maternity care services, especially skilled assistance at delivery, should focus on addressing those negative attributes of the healthcare system that discourage access and use.

## Background

Ghana is one country in which for the majority of women, the experience of pregnancy and childbirth can still in fact be equivalent to a death sentence, characterized by fear, anxiety, anguish and pain. Ghana has had a persistently high maternal mortality ratio, estimated to range from a low of 200 to a high of 1,300 per 100,000 live births [1]. The 2010 WHO estimates suggest 350 maternal deaths per 100, 000 live births in Ghana [2]. Maternal mortality, which accounts for 14% of all female deaths, is the second largest cause of female deaths in Ghana [3].

At both global and national levels, there are different explanations for the persistence of poor maternal and newborn health outcomes (see [4-9]). But one prominent explanation that has recently been highlighted in the literature is poor and unequal access to skilled maternal and newborn healthcare services [6,8-10]. Globally, about 63% of women receive support and care during birth from a skilled health worker [6]. While in high-income countries coverage of skilled birthing services is almost universal, in Africa and Asia, only 47% and 61% of mothers, respectively, give birth with a skilled care provider. In the case of Ghana, there has been steady improvement in the coverage of skilled birth attendance - from 40% in 1988 to 55% in 2010. Despite this improvement, 45% of births are still delivered at home without any form of skilled care, although there are significant regional variations [11].

Since the adoption of the Millennium Development Goals (MGDs) in 2000 – of which goals 4 and 5 aim to improve maternal, newborn, and child health - one policy action area felt to be of particular importance to the reduction of maternal and neonatal mortality is to increase the proportion of women accessing and using skilled maternal healthcare services, especially delivery with skilled health professionals in attendance. It is in this regard that the government of Ghana, in 2003, pioneered and is implementing a maternal healthcare policy that provides free maternity care in all public and mission healthcare facilities. Ghana’s free maternal healthcare policy is premised on the notion that financial barriers are one of the most important causes of low and inequitable access to, and use of skilled maternity care services [12]. The policy therefore aims to reduce financial barriers to access and improve access to and use of skilled delivery services [1].

While a number of studies have suggested that the implementation of the policy of free maternity care has largely eliminated financial barriers to access [12-15], Ghana continues to register strikingly high maternal mortality rates as well as low levels of skilled maternal healthcare services accessibility and utilization, prompting the minister of health to describe maternal mortality as a ‘national emergency’ during the 2008 Ghana Annual Health Summit. One recent World Bank study also suggested that Ghana is off track to achieving the MDG 4 and 5 targets despite implementing the free maternity care policy [16]. The same study further highlighted the fact that among countries with similar levels of income and health expenditure, Ghana performed worse than average with respect to neonatal, infant, under-five, and maternal mortality.

The stall in Ghana’s progress towards improving maternal and newborns health and the gaps in the continued use of maternity services from a skilled provider suggest an urgent need for further research into the factors other than money that might be inhibiting access to, and use of services. Besides, understanding the remaining barriers to access within the context of Ghana’s free maternal healthcare policy forms an important first step towards the establishment of comprehensive policies for the reduction of maternal and neonatal mortality [17]. The objective of this paper is to explore health system factors that inhibit women’s access to and use of skilled maternal and newborn healthcare services in Ghana despite these services being provided free.

## Methods

### Study design

The qualitative data reported in this paper were extracted from within a larger, original study that the authors conducted to examine the effects of Ghana’s free maternal healthcare policy on women’s maternity care seeking experience, equity of access, and barriers to accessibility and utilization of maternal and newborn healthcare services. The design of this larger study followed a mixed methods approach; involving analysis of a nationally representative retrospective household survey data in combination with qualitative exploration using data generated from anthropological research techniques of focus group discussions (FGDs), key informant interviews (KIIs), case studies and structured field observations. While other aspects of the qualitative data from the larger study have been analysed, written up and submitted for peer-review by other journals, this paper focuses on and report findings from an aspect of the qualitative study that explored aspects of Ghana’s maternity care delivery system that discourage use of skilled care.

### Study setting

Empirical research was conducted in Ghana during a total of 6 months between November 2011 and May 2012 in a total of 6 purposively sampled communities namely, Kuntanase, Abono, and Piase in the Bosomtwe district of the Ashanti region; and Mpaha, Sankpala and Tidrope in the Central Gonja district of the Northern region. We chose Ghana for this research not only because maternal health is seen as a ‘national emergency’, but also because Ghana presents an interesting ideal case study. It is one of only a handful of countries in Sub-Saharan Africa to have actively started implementing both universal maternity care and health insurance policies at the national level. The economic and political conditions in Ghana also make the country an interesting case study. Ghana is situated within the predominantly economically marginalized and politically unstable region of West Africa, but forms an exception. Relatively, Ghana is a small fledgling multicultural, multi-ethnic, multi-religious, and multi-party constitutional democracy, characterized by vibrant civil society activism and media pluralism. It is politically stable; a rebasing of its economy in November 2010 saw the country leap into the category of lower-middle-income countries [16]; it also recently started producing oil in commercial quantities; and is often touted as one of the most politically and economically progressive countries in the region. Although Ghana has since 2013 been experiencing economic deterioration - with some private healthcare providers including the Christian Health Association of Ghana (CHAG) cancelling their contracts with the National Health Insurance Scheme due to heavy indebtedness, thereby forcing all patients whether insured or not to resort to the old cash-and-carry system – the country is still often considered ‘an example of global good practice’ [16,18]. Despite the ‘exceptional status’ Ghana enjoys in the sub-region, maternal, neonatal and infant mortality ratios have remained persistently high.

Apart from capturing a divide between a relatively destitute northern Ghana and a relatively prosperous southern Ghana, we chose the six communities to provide a diversity of social and health situations that are largely representative of the country, Ghana (Table 1). For instance to capture any differences that may exist between urban and rural communities as well as communities with at least one government or mission healthcare facility and those without, we selected Kuntanase to represent urban communities, Piase, Sankpala and Mpaha to represent rural communities with health facilities, and Abono and Tidrope to represent rural communities without any health facility. Although the six communities demonstrated variable levels of performance on maternal and newborn health indicators, access to, and use of antenatal, delivery, post-delivery and newborn care services were mostly below the national averages (Table 1).

**Table 1:** **Basic characteristics of the study communities**

	**Bosomtwe District**				**Central Gonja District**			
**Community characteristic**	**Piase**	**Abono**	**Kuntanase**	**Entire Bosomtwe District**	**Tidrope**	**Mpaha**	**Sankpala**	**Entire Central Gonja District**
Population (2010)	2,772	1,467	34.682	99,964	1,025	4,126	1,526	110,576
Number and type of health facility (2010)	Health centre (1)	0	Hospital (1)	Hospitals (3), Health centres (3), Clinics (7), Maternity Homes (3)	None	Health centre (1)	Health centre (1)	Hospital (1), Health centres (5) CHPS zones (11)
Number of medical doctors (2010)	0	0	2	10	0	0	0	1
Number of midwives (2010)	1	0	3	38	0	0	1	9
Number of general nurses (2010)	0	0	7	65	0	0	0	6
Number of community Health nurses (2010)	2	0	11	25	0	5	4	37
Number of pharmacists (2010)	0	0	1	3	0	0	0	0
Number of dispensary technicians (2010)	0	0	3	14	0	1	0	2
Distance to nearest health facility (km)	1	12	1	n/a	13	2	1	n/a
Doctor to patient ratio (2010)	1: 9,997				1: 110,576			
Nurse to patient ratio (2010)	1: 1,111				1:2,572			
Number of maternal deaths (2010)	n/a	n/a	n/a	5	n/a	2	0	28
Number of under-5 deaths (2010)	n/a	n/a	n/a	25	n/a	10	4	25
Skilled delivery (%) (2010)	56	n/a	74	54.3	n/a	18.1	22.2	14.4

### Ethics

We obtained clearance from the University of Oxford Social Sciences and Humanities Inter-divisional Research Ethics Committee (Ref No.: SSD/CUREC1/11**-**051), and the Ghana Health Service Ethical Review Committee (Protocol ID NO: GHS-ERC 18/11/11). In addition, both informed written and verbal consent were obtained from all research participants. In all cases, consent was obtained after it was thoroughly explained to participants that their participation was entirely voluntary and that information obtained will be used for the purposes of this research only. To thank research participants, we bought biscuits and soft drinks to refresh them.

### Research participants

The research participants were drawn from a population of pregnant women and lactating mothers from the six research communities, and from health personnel from the two regional and district health directorates. For the purposes of this research, we classified participants into ‘Women’ and ‘Healthcare Providers’. ‘Women’ here refers to women who were pregnant at the time of this research or had given birth between January 2011 and May 2012 in the six study communities. Healthcare providers comprised health professionals (doctors, nurses, midwives, healthcare managers, and health policy makers or implementers) from health facilities in the study communities, district and regional health directorates, and Ghana Health Service at the national level. The rationale for interviewing diverse actors at multiple levels was to allow for an exploration of multiple perspectives.

In all, 205 participants took part in the study. Of this number, 90% (185) were pregnant women and lactating mothers. The remaining 10% (20) consisted of healthcare providers.

### Sampling and recruitment procedures

Our strategy for recruiting research participants involved both probability and non-probability sampling procedures. For all research participants under the ‘healthcare providers’ category, a purposive sampling technique was used. This was a judgmental selection based on the participant’s knowledge of the subject of study, the participant’s role in the selected health institutions, and our evaluation and/or perception of the relevance of that role and knowledge to the research topic [19].

For the women however, a simple random sampling procedure was used. The actual sampling and recruitment procedure involved four main steps. First, we enumerated all pregnant and lactating mothers in each of the study communities using a five-item short questionnaire that we designed. The questionnaire asked whether a woman was currently pregnant or had given birth since January 2011, the name of the woman, age and house number/name. Second, after the listing was completed, we randomly selected the required number of participants from the pool of names in each study community. We predetermined the required number of participants (5% of the total enumerated population of pregnant and lactating mothers of each study community). We believe sampling 5% of the enumerated population in each community yielded sufficient numbers of participants whose views on the research topic could fairly approximate the views of the majority of women in the community. Third, the randomly chosen participants were further randomly allocated to either focus group or in-depth interview. Finally, we took the randomly selected names to the various communities wherein the research was introduced and the selection procedures thoroughly explained to each of the randomly selected women. Thereafter, the women were invited to participate in the study. Where any of the randomly selected women was not available or declined to participate in the study – and there were only 2 of such cases – we repeated the selection process to get a replacement.

We acknowledge that the emphasis of qualitative research is not always on generalisation hence randomisation might not be a necessary requirement [40]. However, we used simple random sampling in this study as a pragmatic and ethical strategy to assure justice by using a fair and transparent sampling procedure that ensured that every pregnant and lactating mother in the study communities had a fair chance of taking part in the research. In fact, the idea of chance - which was embedded in our sampling procedures - helped to eliminate questions about why one woman was included and another excluded from the study.

### Data collection methods

Focus group discussions and key informant interviews were the main data collection methods. We adopted focus groups partly because we believe it had the capability to reproduce women’s experiences of seeking maternity care in a normal peer-group interpersonal exchange. Six (6) focus group discussions – one in each community and involving a total of 104 pregnant women and lactating mothers - were completed. Groups consisted of 17–24 participants. This difference was mainly due to differences in the sizes of the target populations. All focus groups were held in the study communities, at venues chosen in consultation with participants and community gatekeepers. Each focus group lasted 1.30 to 2 hours, and ended when a point of saturation was reached i.e. when no new ideas and issues seemed to arise. All discussions were conducted in the local dialects – *Twi* in Kuntanase, Abono and Piase; *Dagbani* in Sankpala and Tidrope; and *Gonja* in Mpaha. This is not only because the literacy [written or spoken English] rates are low among the study participants, but also because we wanted to ensure that the interview language was not a barrier to effectively exploiting the full benefits of focus groups.

Because our knowledge of the interview language was limited, we engaged one female research assistant from each community to facilitate the discussions. Prior to the interviews, these research assistants were trained for three days. The training took the form of classroom lectures on the objectives of the research, a question-by-question explanation of the content of the topic guides, instructions on field and interviewing procedures to be followed, and instructions on how to ask sensitive questions. To reinforce the training interviewers had received and to reduce potential bias in questioning, the training also involved mock or practice field interviewing, during which each of the research assistants interviewed 3 women using the relevant interview language. These pilot interviews were transcribed and translated into English by independent language translators. The results of the pretest suggested that all research assistants performed well in terms of asking questions in the correct way. Where any errors were detected, they were discussed with the appropriate interviewer to ensure that the same errors did not reoccur in the main interview.

To further explore the research question, we conducted key informant interviews to complement the focus groups. The need for a mixed data collection technique in the social aspects of disease and health research has been widely discussed [20]. In particular, it has been argued that people may not necessarily tell the truth in any objective sense when it comes to sensitive issues such as health and disease within a group context [21]. To overcome this, we complemented our focus groups by conducting key informant interviews. Key informant interviews were also used because it was difficult to organise focus group discussions with healthcare providers. This was largely because it was extremely difficult to get a group of healthcare workers to agree on a common time period to hold FGDs. This was due to the different duty-schedules of individual healthcare workers. This was overcome more appropriately by conducting individual interviews. One major advantage of the method was its ability to address sensitive issues such as personal experiences and perceptions with regard to distribution, accessibility to, and utilization of maternity care services.

In all, a total of 101 key informant interviews were completed – 81 with pregnant and lactating mothers, and 20 with selected healthcare providers. The distribution of the 81 women across the six study communities was 21, 11, 15, 12, 13, and 9 in Kuntanase, Abono, Piase, Sankpala, Mpaha, and Tidrope respectively. Interviews lasted 10 to 15 minutes. All interviews with women were conducted in *Twi*, *Dagbani,* and *Gonja.* Interviews with healthcare providers were however conducted in English.

### Research instruments

In all focus groups and key informant interviews, we used an open-ended thematic topic guide. The instrument was designed to ensure that similar themes were covered in each discussion or interview. The instrument however had built-in flexibility that allowed questioning to flow naturally while permitting us to pick at random and probe more on any pertinent but unexpected issues that arose during the interview process. The instruments focused primarily on exploring women’s experiences of seeking or not seeking maternity care services, issues regarding coverage, utilization and access, women’s interaction with maternal and newborn healthcare services, the barriers to access and use of services, and perceptions of in/equities in maternal health services accessibility and utilisation. In addition, a more structured questionnaire instrument that sought to collect specific socio-demographic information as well as elicit specific response (e.g. did you or will you give birth at the hospital, clinic or home?) from all women was designed and administered individually to all women.

To ensure that the instrument was reliable, we engaged in a continuous review of the questions and the interview process to ensure that they were eliciting the right answers to the right questions. This proved valuable in enabling us to reframe questions, clarify and use more appropriate or easily understandable concepts as the research progressed. All discussions and interviews were tape-recorded alongside hand-written field notes.

### Data analysis

To ensure that the qualitative data analysis process was methodical and transparent, we followed the Attride-Stirling’s thematic network analysis framework [22]. Several steps were followed before the analysis proceeded to an interpretative phase in which the networks were connected into an explanatory framework consistent with the text. The first step involved transcription and reading of transcripts and field notes for overall understanding. During and after qualitative data collection, the first author and three other language specialists - *Twi, Dagbani* and *Gonja* – transcribed all tape-recorded interviews. The first author then immersed himself in all transcripts and interview notes through reading and reviewing for overall understanding and comprehension of meaning. This first step was completed with a separate summary of each transcript outlining the key points participants made in response to the questions.

Once the data was reviewed and a general understanding of the scope and contexts of key experiences was attained, the interview transcripts were exported to NVivo 9 qualitative data analysis software, where the data was both deductively and inductively coded. Codes, according to Miles and Huberman [23] are labels, which are assigned to whole or segments of transcripts and interview notes to help catalogue key concepts while preserving the context in which these concepts occur. Coding provided us with a formal system to organise the data, uncovering and documenting additional links within and between concepts and experiences in the data. Data coding continued until theoretical saturation was reached. This was a point where no new concepts emerged from successive reviewing and coding of data [24]. At this stage, the code structure was deemed complete and then applied to develop and report themes. Themes simply captured something important about the data in relation to the research question, and represented some level of patterned response or meaning within the data set [25].

Finally, all the themes identified in the previous steps were assembled and a thematic chart was drawn to reflect basic themes, organising themes, and global themes (Table 2).

**Table 2:** **Thematic network analysis framework (from codes to global themes)**

**Codes**	**Basic themes identified**	**Organizing themes**	**Global themes**
-There is joy in pregnancy & childbirth	1. Pregnancy & childbirth are fulfilling biological functions women perform	1. Pregnancy & childbirth is role fulfilment, self actualization and empowerment	Women’s experiences of pregnancy & childbirth
-Women feel accomplished in giving birth safely.			
-A woman can die while pregnant or giving birth	2. Pregnancy & childbirth is dangerous	2. Pregnancy & childbirth can be a dangerous event	
	-you either die or live		
-Pregnancy & childbirth is an anxious phase of woman’s life			
-Pregnancy makes women highly dependent on others			
-A Pregnant woman needs care, love and empathy to be able to deliver safety	3. Care during pregnancy is important for safe delivery	3. Women want skilled attendance at birth	
-Women should go to hospital when pregnant	4. Hospital delivery is good		
-It is good to deliver in a hospital			
-Midwives can help to deliver women safely			
-Some places do not have health facilities	5. Unequal availability and/or distribution of services gives rise to differential access		
-There are no nurses/midwives/doctors at some health facilities		4. Health system failures	Barriers to access & equitable access
-Services are far away	6. Long distance travel & difficulties with arranging transport to health facilities discourage access		
-It is difficult to travel			
-Caregivers do not ask how women feel	7. Poor services quality discourage women from accessing and using services		
-There is overcrowding in maternity wards			
-Maltreatment and scolding is dehumanizing			
-Caregivers want women to obey instructions without question	8. Lack of choice in health facilities is a disincentive to use of skilled care		
-Poor communication between women and caregivers leads to misunderstanding	9. Women’s distrust in the ability of health care institutions & professionals reduces their willingness and ability to access skilled care		
-Women do not believe their problems can be solved by some caregivers/health facilities			
-Nurses are disrespectful	10. Culturally and linguistically inappropriate care and health information does not promote effective communication between caregivers & women		
-Caregivers disrespectful and disregard women’s preferences and cultural values relating to pregnancy and childbirth			
-TBAs advise women about what to do in an obstetric emergency, but health system does not recognize and rewards TBAs	11. Lack of recognition of the role of TBAs by the health system breeds mistrust and discourage cooperation even in times of obstetric emergnecies		
-There is privacy in homebirth but not in hospitals.			

To ensure that the thematic chart reflected and supported the data, we went through the data segments related to each basic, organising, and global theme. Where necessary, refinements were made. In total, 23 codes were identified. These were grouped into 11 basic themes that were further clustered into 4 organising themes, and 2 global themes (Table 2). These global, organising and basic themes form the structure of our findings and discussion section. Where appropriate, we use verbatim quotations from interview transcripts to illustrate responses related to relevant themes.

## Results

### Basic characteristics of the women participants

Our study sampled women who have had personal experience of pregnancy and childbirth, and who were fully qualified to have benefitted from maternity care interventions such as the free maternity care policy. The ages of these women varied between 20 and 45 years. The majority of the women had no formal education. A few of the women were unemployed while most were engaged in diverse occupations such as farming, trading, hairdressing, dressmaking, and teaching. Several of the women were also married or living with a partner. The majority of the women had between 1 and 3 children.

Our research participants’ accounts in relation to health system factors that discourage use of skilled maternal and newborn healthcare services in Ghana converged on a number of specific common themes, which are explored below.

### Childbirth and women’s maternity care experiences

Discussions and interviews with women show that childbirth is of special value to men and women in Ghana, whether they are married or not. A woman needs to have children to ensure the perpetuity of her own lineage in a matrilineal society, or that of her husband’s in a patrilineal society in Ghana. For this reason, not only is there joy in pregnancy and childbirth, but also most women spoke of pregnancy and childbirth as role fulfilment, self-actualisation and empowerment.

*For me, I was really happy when I became pregnant. It made me felt fulfilled and accomplished because people now respect me more* (Lactating Mother, FGD, Tidrope).

Because of the importance of childbirth in the life of a Ghanaian woman, several women reported that a pregnant woman needs care, love and empathy to be able to deliver safely. In this regard, there was a general sense of awareness among women about the potential benefits of seeking skilled antenatal care (ANC), delivery care (DC) and postnatal care (PNC) services from a health facility. ‘I believe it is important for every pregnant woman to go to hospital to check their pregnancy’ (Lactating Mother, FGD, Sankpala). Indeed, coupled with the numerous maternal health education campaigns that are being undertaken by the Ghana Health Service, the birth place choice of many Ghanaian women appears to be shifting from the home (where skilled-birth attendants are not available) towards formal healthcare institutions (where skilled-birth attendants are likely to be available). In Sankpala for example, skilled delivery rose from 18.1% in 2009 to 22.2% and 36.1% in 2010 and 2011 respectively [17]. During the same period, skilled delivery in the entire Central Gonja district rose from 13.4% in 2009 to 14.4% and 24.3% in 2010 and 2011 respectively. In Kuntanase, skilled attendance at birth has similarly risen from 68% in 2009 to 74% in 2010.

But while many women spoke of the need to have children and the joy that childbirth brings, there were also stories about the grimmer side of pregnancy and childbirth. Women reported that pregnancy and childbirth was dangerous as ‘you either die or live’. It is the combination of the need to procreate, the joy and fulfilment that childbirth comes with, and the fear that one might die in the process of giving birth, that sometimes warrants care-seeking. At the same time however, the majority of women reported how the organisation and delivery of maternity care services was making it extremely difficult or impossible for some women to access and use these services. While several of these accounts were characterized by both negative and positive experiences, negative experiences dominated women’s daily encounters. In both their collective and individual response, these women were unanimous that these negative experiences were not only endemic under the current free maternal healthcare policy regime, but also that they were so disabling for the majority of women that some women now prefer not to seek any care during pregnancy and labour, or opt for alternative care such as care provided by traditional birth attendants (TBAs). In what follows, we present women’s accounts about how these different health system factors inhibit their access and use of maternity services.

### Service availability, distribution and access

For women to have the ability to access and utilise maternity care services, there must be adequate supply of these services [26]. A fundamental assumption of Ghana’s free maternal health policy is that an adequate supply network of skilled care services are available and if only such services could be made costless or affordable, all women would access and use them. Interviews with women and healthcare providers, which were corroborated by systematic observations, reveal that this is not the case in many districts and communities in Ghana. Our interview data suggest that while the policy of free maternity care should in principle benefit all women, in reality the success of the policy depends on access to health facilities which many women, especially poor rural women, lack because of long distance or transport and other opportunity costs constraints during referrals or emergency situations. For example, in Tidrope and Abono, there is no health facility hence the provision of maternal health services is not readily available. The distance from any of these communities to the nearest health facility is approximately 12 km. In these communities, pregnant and lactating women tell of how a consideration of the prohibitive costs involved in travelling to access or utilise birthing services often lead several families to decide in favour of non-access and use or resort to self-medication. A lactating mother illustrates:

*You see, in this community there is no doctor or nurse whom we can easily go to for help. Sometimes, we really want to go and check if all is well with our pregnancies or babies. Some women even want a doctor or nurse to deliver them, but look at where the hospital is…very very far away. Even some times when we want to go, how to get car is a problem…because of the long distance, many women just stay at home (Lactating Mother, FGD, Abono).*

Indeed, available anecdotal data (see Table 1) as regards the availability and distribution of healthcare facilities and healthcare personnel largely corroborated the women’s accounts. For instance, whereas the doctor to patient ratio in 2010 was 1: 9,997 in the Bosomtwe district, the Central Gonja district had 1:110,576. Similarly, the nurse to patient ration was approximately 1: 1,111 in the Bosomtwe district compared to 1: 2,572 in the Central Gonja District. These ratios are far above the WHO recommended doctor-to-patient ratio of 1: 6000 and nurse-to-patient ratio of 1: 500.

Interviews with healthcare providers on the subject of service availability and distribution also corroborated the accounts several women gave. However, most healthcare providers related the problem to resource constraints and the geographic spread of communities.

*The thing is, not all the communities in this district have health facilities. But the problem is that the resources to do this are limited. Even if we do have resources, it is not feasible to have a health facility in every community due to the scattered nature of settlements Male Healthcare Provider, KII, Mpaha).*

Interviews with women and healthcare providers in Mpaha, as well as our own systematic observations in Buipe, graphically illustrate the effect of the unequal distribution of services on access to birthing care. Women and healthcare providers in Mpaha alike reveal that the absence of a midwife, coupled with the incompetence of existing available nursing staff to manage deliveries was a major factor impeding access to skilled care at the Mpaha health centre. At the Buipe rural clinic, we observed that the number of nurses attending to women who had come for ANC was not only very limited, but also they [nurses] were regularly intermittently called upon to attend to other patients presenting with different ailments. A healthcare provider at the Buipe rural clinic summed the issues thus:

*You are doing research on maternal health access…you have been here, you have seen our staff strength and you have seen the kind of resources and equipment we are working with. How can we ensure that all women have access to good care? Just look at me, I am the only midwife, and look at all the women sitting outside, how can one person take proper care of all of them. Sometimes, I believe the women are right for not coming to us (Female Healthcare Provider, KII, Buipe).*

### Quality of care and access

Interviews with women also indicate that the quality of maternal healthcare offered at health facilities and the time it takes to receive it from a skilled provider is another more important factor determining access and utilisation. Discussions and interviews with women in Kuntanase, Piase, Sankpala and Mpaha - where skilled maternal healthcare services are readily available - showed that women’s experiences of overcrowding and delays in maternity wards, inefficient referral systems, substandard care, and lack of critical healthcare staff to provide needed care have led to widespread dissatisfaction and unwillingness to access and use skilled care despite these services being free. A lactating mother makes the point thus:

*I don’t know whether I should say the apimfuo insurance [the free maternity care policy] has come to help heal our sickness or kill us. When we used to pay, the doctors were taking good care of us. But now that it is free, you go to hospital and they tell you there is no medicine because the government didn’t give them more money…so now most of us will even travel far to get good care (Lactating Mother, KII, Kuntanase).*

Another young mother reports:

*Why I didn’t go to hospital? The problem is even if I go, the people are many…everywhere is crowded and I have to wait for long hours or even a whole day. Sometimes too you’ll go and they [referring to nurses] will tell you that the midwife is not there. If they decide to help you too, they will just rush and say go home…no blood testing, no medicine, nothing! I don’t know…but I think because the nurses no longer collect money from us, they are very reluctant to help us (Lactating Mother, FGD, Piase).*

Our observations and interviews with healthcare providers reveal that delays and overcrowding were often exacerbated by the fact that people came at random or without prior appointment to seek care in healthcare facilities. This was because there is no booking system in place to enable women book appointments prior to visiting healthcare facilities. Several women reported that standards of care were particularly poor in government healthcare facilities compared to private facilities. Thus the idea that, government healthcare centres offer low quality maternal healthcare services was widely cited for both non-use of the services and a high level of preference for services offered by non-government healthcare providers. At both Buipe rural clinic and Sankpala health centre where we were able to systematically observe the doings of ANC clinics, overcrowding and long delays were evident. Women were found sleeping on benches or sitting on cemented floors for long hours before they were hurriedly attended to. It is within the above context that some of the women interviewed for this study were willing in some instances to travel further in order to access quality care.

While many women believed the quality of care they were receiving under the free maternal healthcare policy was generally poor, a number of the healthcare providers interviewed gave conflicting answers. One male healthcare provider puts the quality of care problem this way:

*I think our pregnant women are smart…they know that they can benefit from ANC or DC services and therefore they come to the clinic/hospital. At the same time they know that in an ANC clinic or a labour ward in a health facility they are hardly better off, so they choose not to come or simply deliver at home (Male Healthcare Provider, KII, Accra).*

Others however believed quality of care had actually improved. Even where they acknowledged that the care quality was sub-standard, they often justified it by reference to lack of monetary resources to purchase medical equipment and drugs or limited number of health staff or the increased workload wrought by the free maternity care policy itself.

*One of the good things about the free maternity care policy is that we have been able to considerably improve the quality of the services we now provide. As you may know, the policy itself has led to more patients coming to the hospitals… sometimes workers and the facilities are over stretched (Female Healthcare Provider, KII, Kumasi).*

While it was not possible for us to independently determine whether the overcrowding, delays and poor quality of care women reported receiving were caused by the free maternal healthcare policy, our observations, coupled with interviews with nurses and midwives as well as reviews of secondary literature, suggest that the quality of maternal healthcare may indeed be deteriorating.

### Trust and access

Aside from imbalances in the distribution of health resources, perceptions of declining quality of care, and a healthcare system that is technocratic and impersonal, one of the recurrent themes from our interview data is the issue of trust in both the healthcare system and the providers of care. A pre-requisite for effective patient-provider interaction is the patient’s trust that the provider is knowledgeable and motivated to provide the best care available [32]. In the districts where this research took place however, women’s narratives and testimonies - which were largely, corroborated by healthcare workers – show that increasing distrust in the knowledge, skills, practices and competence of maternity care facilities and caregivers (nurses and midwives) and the safety and efficacy of the ANC, delivery and post-delivery care they provide to women, is undermining access.

*I think many women are not going to hospitals because of the people there…I mean the nurses. Some time ago we had good nurses and midwives at the hospital… they understood what it is like to be pregnant or be in labour because they themselves have been through it. But nowadays, all those old nurses are not there… the place is filled with small, small, small nurses and midwives who don’t know how to take care of pregnant women because they have no experience of giving birth. So when you go to them for help, you are in trouble… they don’t take proper care of you (Pregnant Woman, FGD, Tidrope).*

A lactating mother also said:

*You see those young nurses and midwives at the hospital are not doing well at all. Normally they feel that they know book, but in fact they don’t know how to deliver a woman properly because they all have never given birth. One of the midwives…she is younger than my daughter, how can she deliver me? How can they help you deliver when they have never even been pregnant themselves? Look… Antie Maggie [referring to a TBA] who doesn’t even know book, when you are in labour and they call her to come and deliver you, you will see how she will run to you…even the way she will talk to you…you will not even be aware and the baby will just come out (Lactating Mother, FGD, Kuntanase).*

Another participant adds:

*Are you asking why I didn’t go to check my pregnancy? Some of the nurses are to blame…because they don’t take good care of us like some time ago (Pregnant Woman, KII, Abono).*

Interestingly, women’s lack of trust in caregivers was based on other criteria than strict evaluation of whether caregivers have been adequately trained to obtain the required clinical skills to give maternity care. Rather, women often made a distinction, albeit tacitly, between having experiential knowledge of pregnancy and childbirth on the one hand, and acquiring book knowledge about pregnancy and childbirth on the other. Women argued that although book knowledge is necessary, a midwife ought to have had a personal experience of pregnancy and childbirth. Such a midwife, the women suggested, did not only possess the ‘technical know how’ of pregnancy and childbirth particularly in times of complications, but also is well positioned to empathise. Empathy and sharing of personal experiences are two elements that all the women in this study believed were important, especially for safe natural delivery. However, the women noted that most of the young midwives possess only book knowledge, but are unable to empathise or relate to their [women] experiences. It is therefore the absence of empathy and sharing in the maternity care practices of young female midwives more generally, and male midwives in particular, that makes older female midwives and TBAs highly preferred.

Interviews with frontline midwives/nurses and healthcare personnel in senior management capacities also reveal their awareness of women’s diminishing trust in the healthcare system.

*What we have heard is that, the women don’t come to the health facilities because they feel we are young and have no experiences of childbirth…that we only have book knowledge. To some extent, the women are right. Look at this health centre we serve about 64 communities, and yet there is no midwife. So many times we the community health nurses are the ones conducting ANC, deliveries and post-delivery clinics. But we all have not had training in midwifery, so sometimes there are some problems we are unable to solve. Even there is a lot of new equipment in our stock that we don’t use because we don’t actually know how to use them. So, when we try our best and still there is a problem and we refer the patient to Tamale teaching hospital or Kintampo, the community people say we don’t know anything (Female Healthcare Provider, KII, Mpaha).*

District and regional health directors as well as public health nurses were similarly aware of this trust issue. But they explained that they were caught between scarcity/necessity and rejection at the same time. On the one hand over 50% of the already limited qualified midwives are retiring from active service. This has necessitated the training of young midwives. On the other hand however, these young midwives are facing rejection in their various healthcare facilities by the very communities they are suppose to help.

*We know that many women prefer the older midwives because of the perception that some of these young midwives have no experience of childbirth or that they are not friendly enough. So if the older midwives are not there, the women choose not to come to the health facility. But, you see we are short of midwives already and there is urgent need to train more so they can take over from the older ones who are leaving the system. (Female Healthcare Provider, KII, Tamale).*

That there is a trust problem in Ghana’s maternal health system, which is increasingly undermining efforts to ensure increased and equitable access to skilled care is therefore no exaggeration. What is unclear from our interviews and discussions with women and healthcare providers however is the source of this distrust. Few healthcare providers suggested that distrust in the care system might have existed prior to the implementation of the free maternity care policy. For the majority of women in this study the period of free maternity care in Ghana is also a period of widespread distrust in health facility-base maternity care. Both women and healthcare providers’ accounts suggest that the marginal increase in demand for skilled care services initially occasioned by the implementation of the policy, and the concomitant training and deployment of young midwives to both augment and replace aging midwives, may have aggravated the trust problem.

### Intimidation, choice, and access

The implementation of free maternal healthcare policy in Ghana has been accompanied by a progressive and aggressive medicalisation of the human reproductive process – from fertility control, family planning and pregnancy management, to medical interventions during childbirth and postpartum period. Although this is generally seen as essential to reducing maternal and neonatal mortality in Ghana, focus group discussions and interviews with women show that many have found this process intimidating and choice restricting. Consequently, and as a way of both resisting this progressive medicalisation and reclaiming control over their reproductive health, many women are not accessing and using the skilled birthing services that are provided at healthcare facilities.

During interviews, women spoke passionately about the naturalness of pregnancy and childbirth. Many believed that, as much as possible, there should be limited ‘unnatural’ intervention or interference during pregnancy and childbirth. It is in part the idea of the naturalness of pregnancy and childbirth that causes childbearing women in this study to be apprehensive of any interventions believed to be ‘unnatural’, including caesarean section. Women particularly emphasised the need for choice in terms of place and position of birth as well as the desirability of limited intimidation during pregnancy and childbirth. These were thought to greatly facilitate the natural processes of childbirth. Many however lamented that the care that healthcare facilities and healthcare providers offer were often less choice enhancing and less stress-free.

First, intimidation. For women, the hospital – itself an unnatural environment - with all its modern technological edifices is a rather intimating and disabling environment to give birth. One woman said:

*I don’t like the hospital. All those funny funny machines that the doctors use make me fear the place. When I had my first son, it was in the hospital and the doctor put this huge thing … it was like a big scissors [referring to forceps] into my private part. It was terrible! Also, the smell of medicine and the screaming of other patients…it just makes me sicker. So unless I am really suffering or dying, I prefer not to go (Pregnant Women, FGD, Kuntanase).*

A lactating mother adds:

*I didn’t deliver at the clinic because I wanted to be at a place like my house where I can feel free to spit, urinate, cry and do anything that I feel like doing. In the hospital, the nurses will not allow you or they will even insult you, and this makes it hard for you to relax and deliver well (Lactating Mother, FGD, Sankpala).*

But intimidation is not the only thing that prevents women from accessing and using skilled care services. The restrictions placed on their ability to decide on where and how to give birth, is another reason. Women believed that nurses, midwives and doctors limited their choices related to labour and birthing as well as who should witness their birth. For example, women expressed their preference for delivering in an upright position including sitting on a stool or chair, squatting or sitting upon the side of the bed or being held by other women that come to labour. Women who have had home delivery and therefore the opportunity to choose their birth position noted that delivering in an upright position facilitated birth of the baby as well as removal of the placenta. They however noted that the recumbent position favoured by nurses and midwives contributed to general difficulty of delivery. Despite this, delivering on the hospital bed, usually a flat and non-inclining bed, in a supine or recumbent position is the accepted birth position in most healthcare facilities in Ghana. Similarly, family members and friends are usually not permitted into the labour ward. Yet, many women said they feel more confortable delivering with their family members present to offer support. Since nurses and midwives did not usually allow this in the health facility, they found home birth more confortable.

*For me, giving birth at home is more comfortable. I gave birth at home where my mother, my mother-in-law and other neighbours were available to support and care for me. In the hospital, I will not get all this support (Lactating Mother, FGD, Piase).*

For the women in this study, successful pregnancy and delivery requires that women be given the chance to choose where to deliver and in which position.

### Silent care, maltreatment, and access

Several of the women interviewed also recounted their experiences of how they were chided and scolded for not coming to the healthcare facility early to seek care or when they did not attend ANC clinics or when they used an alternative medicine or when they failed to practice birth control or when they asked the nurses/midwives/doctors questions related to their (women) health or that of their baby. Particular reference was made to nurses, midwives and female health providers in general, as exhibiting poor attitude towards pregnant and labouring women. Others related how they were often offered ‘silent treatment’ and treated like ‘children’ and in some instances threatened with treatment withdrawals if they failed to adhere to advice and instructions from healthcare workers. One participant relates her experience thus:

*I have made up my mind not to go to that hospital [referring to Kuntanase hospital] again. Because, the nurses are suppose to know and tell me what is wrong. But the last time I went, they told me to go spit my saliva into a container and bring, I did and they ask me to go and bring my urine too. So I asked the nurse what was wrong. She didn’t say anything but asked me do as I am told. But I asked again, and then she became angry and started to say I was a villager and that I didn’t know anything. I was embarrassed and became very angry because everybody at the clinic was looking at me and laughing. We are human beings with emotions and feelings, but they never ask how we feel…they don’t…they really don’t care (Pregnant Woman, FGD, Kuntanase).*

Women reported that scolding, maltreatment and silent treatment were particularly worse in government healthcare facilities than in private facilities - a phenomenon, women said, discouraged them from seeking skilled care services when in need. Discussants said that health workers in private health facilities were more hospitable than their counterparts in state-run services that often abused patients. In this way, ownership of a health facility emerged as an important determinant of the nature of treatment received. In Sankpala health centre and Buipe clinic where we observed the conduct of antenatal and post-natal clinics, several medical procedures including blood pressure and temperature check-ups, weighing, and immunisation were conducted on women and their babies without any accompanying explanations of the purpose of the medical procedure or its functions. Women also regularly stood up when responding to questions from nurses. At the same time, nurses seemed unperturbed by the precarious condition of most pregnant women neither did they seem to be mindful of the fact that some of these women were far older than they (nurses) were. Note that age within most Ghanaian societies has social significance. It confers on older persons a right to particular treatment, including respect, especially from younger people. As we later learnt, nurses’ failure to respect elderly women at the clinic is one reason why some women choose not to consult nurses and midwives. A few women however revealed that in order to receive the care and treatment they need or avoid getting into conflicts with nurses, they often have to accept the scolding, intimidation and disrespect in maternity wards without openly expressing their feelings.

Most of the healthcare workers interviewed appeared quite aware of what the women called the silent care and maltreatments. However, they either underestimated the effects this has on women’s health-seeking behaviours or justified it by reference to work overload and tiredness that often stressed healthcare workers. Others also justified silent treatment on grounds of language differences between healthcare workers and patients, which sometimes made it difficult for nurses and midwives to effectively communicate health information and treatment procedures to women.

Our interviews with women showed that the lack of open and non-judgemental patient-doctor relationships that allow communication, mutual trust and respect to flow between caregivers and women did have a disabling effect on uptake of services. This is because any time that a woman makes the decision to access and use maternity care services at a healthcare facility, her general expectation is to be able to enter into a positive relationship with the healthcare provider so as to enable her address her healthcare needs and concerns. Women expect warm conversation, sharing, and empathy to develop between them and their healthcare provider. Women also expect that when they seek care at the health facility, not only should nurses/midwives/doctors offer them the necessary treatments but also they [caregivers] should help them [women] to understand their health problems, provide guidance needed for making informed decisions about their health as well as answer their questions or respond to any health concerns they may have. Because most women have limited education, they strongly depend on the oral advice and information from healthcare workers. Unfortunately, several women reported that their expectations are very often not met. Rather, authority and passivity heavily characterise the interactions between healthcare workers and women, especially in the ANC clinic or labour ward. Thus doctor-patient relationships are structured around power and control. This created a significant gap between existing maternal health services and women’s requirements for supportive care. This gap was often compounded by the cultural and functional adjustments women often have to undergo when a visit or referral is made to an unfamiliar health facility setting or healthcare provider. The combined effect of this has been to discourage access to, and utilisation of services.

### Privacy and access

Women also reported that under the current maternal and newborn healthcare regime in Ghana, hospital and clinical structures and practices make it extremely difficult to maintain privacy when treatment is being received or when discussing their healthcare concerns with nursing staff.

*You see, I can get up and go to Antie Maggie [referring to a community TBA] to check my pregnancy and nobody in this house will know. But if I go to the clinic, there are so many other people sitting there. Everybody is listening to what you are telling the nurses…sometimes, there are things you want to tell only the nurse or you want to ask the nurse alone. But because there are other patients, you can’t (Pregnant Woman, FGD, Tidrope).*

One participant also relates:

*I didn’t deliver at the hospital because the first time I gave birth at the hospital…it was not too good for me. I didn’t like the way the place was open. There were other patients, nurses, midwives and doctors in the labour ward. So everyone could see you or hear what you are doing. I think it would be better if every patient had their own delivery room, or even if they can use curtains to cover you (Lactating Mother, FGD, Abono).*

Despite the importance women attached to privacy, interviews with nurses, midwives and healthcare managers showed that ensuring privacy was often not an issue of primary concern. The few who acknowledged the relevance of preserving privacy in ANC clinics and labour wards even countered the argument by highlighting the limitations imposed by infrastructural constraints.

*I think that it is good that we maintain some level of privacy in our maternity wards. But the issue is, when you don’t have space or when you have only one person attending to several hundreds of women, how are we supposed to maintain privacy? We really can’t, unless we choose to take care of few women and ignore the rest (Female Healthcare Provider, KII, Sankpala).*

Indeed, our observations around ANC clinics and maternity wards showed that privacy was neither ensured nor given serious importance. For example, case histories and clinical examination of pregnant women took place in the midst of others waiting to be attended to. This was confounded by nurses’ penchant for interviewing women in loud voices. This made it very easy for those waiting to hear the health problems or concerns of other women. For fear of ones health problems being subjects of gossip, many women said they felt constrained telling nurses all their health concerns within those settings. For these women, the limited privacy within the health facility setting and the way in which caregivers ignored this crucial aspect of care was an important barrier to accessibility and utilisation of skilled care services.

## Discussion

This paper explored health system factors that limit women’s access to and use of skilled maternal and newborn healthcare services in Ghana despite these services being provided free. The paper responds to recent calls for more empirical research to understand the remaining barriers to maternal and newborn healthcare access in contexts where services are provided free of charge [17]. Qualitative findings suggest that several health system factors have acted alone or jointly to inhibit women’s ability to access and use skilled care services. Limited and unequal distribution of skilled maternity care services was found to be an important health system barrier that hindered women’s access to and use of maternity care services. While the mere availability or equal distribution of maternity care services might not guarantee automatic access or utilization for all women, our research with women in the two study districts clearly showed that the unavailability of skilled care services in several communities, coupled with the related problems of long travel distance and time, difficulties with arranging transport, the high cost of arranging transportation as well as the opportunity costs of travelling long distances in order to seek care, constituted a major barrier to access. Our findings here therefore suggest that although removing user-fees has the potential to improve access to skilled care, it is neither sufficient nor appropriate for increasing and sustaining access in some contexts. Therefore the policy debates on user-fees removal ought to proceed beyond broad evaluation of the benefits and/ or impact of user-fees removal towards exploring how best to dismantle and address the multiplicity of access and utilisation barriers other than money in differing contexts. In this regard, we believe a concerted multi-sectorial approach is needed, including improving the availability of physical health infrastructure and human resources as well as addressing disparities in distribution. Also, there will be the need for better planning and the institutionalisation of a booking or appointment regime so as to reduce overcrowding in health facilities and long waiting times before care is received. This of course could be problematic to implement in the short term especially in remote areas where communication and transportation services might be lacking. However, in the long term, healthcare facilities can establish systems in local communities that could be used to facilitate booking before attendance. For instance, community health workers could be trained and equipped with mobile communication devices. These community health workers could then be tasked with the responsibility of receiving booking requests from women in the community and then communicating such requests to the appropriate healthcare facilities.

Poor care quality was also identified as a major health system barrier to service use. Elsewhere, other researchers have drawn attention to bad quality care as a barrier to accessibility and utilization of skilled care services in low-income countries [30,31]. While our study was unable to trace the genesis of substandard care practices within the maternal healthcare system in Ghana, it was clear that the period of free maternity care had seen a decline in the quality of care that women received in healthcare facilities. Indeed, previous research and reports have highlighted the issue of poor care quality. For example, one previous clinical evaluation of hospital-based maternity care in Ghana found that on admission women often received poor care [27]. Our analysis of literature related to the overall performance of Ghana’s healthcare delivery system also suggested that the issue of poor maternity care quality might be linked to a generalised health delivery system problem [28]. For instance, a recent World Bank evaluation of the health sector in Ghana similarly concluded that Ghana’s health facilities do not meet appropriate standards of care: ‘most clinics, health centres and maternity homes did not have appropriate standards for emergency obstetric and neonatal care’ ([29], p.48).

Because of poor care quality, our discussions and interviews with women revealed that in some instances, women travelled longer distances to health facilities where qualitative of care was perceived to be better. That women were willing to commute longer distances to access quality care is particularly interesting because it shows that women sometimes consider the quality of care more than the cost. This is one of the central arguments Thaddeus and Maine [13] made in their well-known ‘three delays model’ of accessing maternity care services. Our findings here are therefore significant in highlighting the fact that poor quality of maternal and newborn healthcare services, real or perceptual, does prevent women from accessing and using care services even if such services are freely available.

If access to skilled care is to be increased in Ghana, there is the need for improvement in care quality. This is more so because it would be unethical to continue to promote access to known bad maternal health services. Besides, the focus in the past few decades on encouraging a shift from home-based to institutional delivery will be misplaced if efforts to improve the quality of care a women receives in a health facility is not improved. Further research to not only assess the quality of maternity care provisioning, but also to develop simple, replicable assessment tools and techniques that could be used to evaluate the quality of care provided at health facilities would be important. The development and use of such tools could enable maternity care providers and healthcare managers to easily identify maternity care facilities that deliver sub-standard or sub-optimal care. This could be important for both making and monitoring the desired levels of improvements.

Women’s distrust in the ability of some healthcare facilities or healthcare providers to properly address their maternity needs was another health system barrier to service use. In our focus groups and key informant interviews, it was clear that where there was erosion of trust or confidence whether in a health institution or a healthcare provider, the legitimacy and scientific and therapeutic authority of such institutions or persons was not only undermined, but also women failed to access and utilise skilled care services offered by such institutions or care providers. This is consistent with one recent qualitative study in Cambodia, which reported that women’s perception of the limited midwifery competences of skilled birth attendants at health centres undermined their decision and willingness to access and use services [33]. Women’s distrust in the skills of caregivers and maternity care institutions in our study appears to re-echo the findings of one previous clinical evaluation panel in Ghana, which reported that the competence and ability of health personnel to deal with obstetric emergencies was inadequate; hence they seemed ill prepared to deal with some of the common obstetric conditions [27]. In addition to improving the midwifery skills and competencies of nurses, there is an urgent need for the Ghana health service to institute trust-building programmes that will enable women to understand that the young midwives can equally take good care of them if they [women] come to them [midwives].

The study also found limited birthing choices and lack of privacy at healthcare facilities to be a major health system barrier. Within reproductive medicine, ‘choice’ and ‘right to choose’ are terminologies very explicitly associated with women’s control over their reproductive decision making. In many high-income countries in the West, a shift from highly medicalised hospital births to women-friendly midwifery approaches to childbirth has occurred during the last two decades [34]. In the UK for example, government recommendations about the choice of maternity care since the early 1990s have been that, the policy of encouraging all women to give birth in hospitals cannot be justified on grounds of safety and that women’s choices should be allowed [34]. Despite this, for several of the women sampled in our study there are limited birthing choices which the modern Ghanaian system of healthcare offered, and this has largely acted to exclude several women who desire some choice and freedom during pregnancy or labour.

By choice, women talked about freedom and opportunity to decide and to disclaim, and to accept and to repudiate. Unfortunately, women said these were all options often denied them under the current maternal healthcare system in Ghana. For example, despite years of advocacy for domiciliary delivery with skilled health personnel in attendance to address women’s special needs, the hospital remains the only sanctioned birthplace where women are expected to be delivered and to be covered by the free maternal health policy. In instances where women have expressed a desire to be delivered at home by a trained birth attendant, healthcare workers do not often support such desires. Similarly, nurses and midwives would usually not go to women’s homes to conduct deliveries even if they are called upon to do so. Rather, midwives expect the labouring woman to be brought into the health facility before help was offered.

It might be true that limiting women’s birthing choices to a health facility setting in Ghana is the best way to improve maternal and newborn health outcomes. An argument for choice would therefore have to take this into account as well as the ability of the health system to effectively promote the particular choice. Indeed, given that current global consensus on improving maternal and newborn health puts emphasis on health facility delivery, it is perhaps out of fashion, if not controversial, to raise the point about where childbirth takes place. However, as findings in this study show, the provision of skilled maternity care services free-of-charge at healthcare facilities only, although may be in the long term interest of birthing women, limited their choices and discouraged access to care.

Women’s experiences of intimidation, unfriendly healthcare providers and cultural insensitivity in healthcare facilities were another set of health system factors that discouraged women from seeking skilled care. Interviews with women showed that the lack of open and non-judgemental patient-doctor relationships that allow communication, mutual trust and respect to flow between caregivers and women did have a disabling effect on uptake of services. This supports the findings of other studies in Africa that showed that bad relational practices of healthcare provides have a negative effect on access to, and equitable use of skilled care [31,35]. Surprisingly, many of the healthcare providers interviewed remained unaware of, or simply ignored the impact of their relational practices on women’s maternity care seeking behaviours. With growing global emphasis on the principle of autonomy (the idea that people should be able to reflect on their own best interests and make decisions for themselves) and patients involvement in healthcare, it is particularly worrying that Ghana’s maternal health system follows the traditional model of doctor-patient relationship in clinical practice where the physician has a paternal role, making decisions about what is believed to be in the best interest of the patient, and the patient is expected to accept treatment or advice without question [36]. As a way to redress the situation and improve women’s experience of skilled care within the formal healthcare system, we recommend the development of a standard guideline - albeit with possibilities for making adjustments to address context-specific issues – that could be implemented across all healthcare facilities to improve the treatment expectant mothers and their immediate family receive at healthcare facilities. We also directly call for changes in midwifery and obstetrical and gynaecological education and practices, including emphasising patient-centred care and behaviour change strategies for healthcare providers and service users, improving both clinical and relational quality of care, and enhancing provider competencies.

Findings also suggest that although many women desired privacy in maternity wards, privacy was neither ensured nor given serious importance. That women desired privacy was largely because of its symbolic and practical importance in the management of pregnancy and childbirth. Within the socio-cultural milieu of the communities in which our research took place, pregnancy and childbirth are phenomena often consigned to the private sphere. The processes leading from conception to birth, and even immediately after birth, are shrouded in secrecy, and all efforts are made to shield the process from the prying public eye. It is within this context that women insisted on keeping what is private, private. It is also in this light that women found the maternal healthcare services offered at clinics, health centres and hospitals lacking in offering choice, protecting their privacy or simply keeping the private, private. In this regard, it will be important for clinical structures and procedures to be restructured to preserve the privacy of women. For instance, in health facilities where private consulting rooms are limited, curtains could be used in open wards to create private small spaces where consultation could be held. This will at least ensure that the consultation process is not held in the open.

In sum, our study has shown that fundamental weaknesses in Ghana’s maternal healthcare delivery system have contributed to poor access and use of skilled care services. This knowledge reinforces the findings of other studies in some parts of Africa that have identified lack of access and inequitable access to skilled maternity care services as a function of poorly functioning health systems [37-39]. Our findings are also unique in challenging efforts that concentrate on providing free maternity care without the needed regard for availability, accessibility, and the quality of care provided. This aspect of the discussion further highlights how a focus on patient-side or demand-side factors can conceal the fact that many health systems and maternity healthcare facilities in low-income settings such as Ghana are still chronically under-resourced and incapable of effectively providing an acceptable minimum quality of care in the event of serious obstetric complications. Ghana’s progress towards achieving the maternal and child health-related Millennium Development Goals would greatly benefit from efforts that address these negative attributes of the healthcare system.

Our findings and recommendations should however be read against the backdrop of certain limitations. The qualitative research reported in this paper was conducted in only six communities and in two districts of Ghana. While focusing on a small number of communities enabled us to gain a more in-depth understanding, we recognise the limitation of generalising the findings to other parts of the country, as it may not be representative of what is prevalent in the entire population. The above notwithstanding, important lessons can be drawn from our findings regarding what is actually happening in practice following the implementation of the free maternal health policy Ghana.

In addition, the fact that the data was collected between 2011 and 2012 is of concern. One can assume that much has happened since then and that conclusions drawn from these data may not capture current utilisation levels. In addition, collecting data through recall of reproductive history generates information that is even older than the date of collection, and this may also be associated with a risk of recall bias. These notwithstanding, we believe the broad patterns of women’s experiences of accessing and using maternal healthcare services are likely to remain. Important lessons can therefore be drawn from the findings in this paper to inform policies that seek to encourage women to use skilled maternity care services.

## Conclusion

The analysis of data from interviews and focus groups with women and healthcare providers in Ghana in this paper suggests that although Ghana’s free maternal healthcare policy appears to have shifted the birthplace choice of a significant number of Ghanaian women from the home towards formal healthcare institutions, the maternal healthcare delivery system in Ghana lack many attributes of a functional healthcare system. The healthcare system is still characterized by limited and unequal distribution of maternity services, poor quality of care, distrust in the healthcare system, difficulties relating to arranging suitable transportation to facilitate efficient referrals, women’s experiences of intimidation in healthcare facilities, unfriendly healthcare providers, cultural insensitivity, long waiting time before care is received, limited birthing choices, and lack of privacy at healthcare facilities. Thus the anticipation of attending an inadequately equipped and dysfunctional health facility, where the potential for further referrals to more distant hospitals with significant costs implications is rife, partly explains why some women in Ghana do not use skilled maternal and newborn health services at health facilities. Even where skilled maternal and newborn healthcare services are available at no or minimal cost, and can be physically accessed, our study found that such services can be unfriendly, socially degrading or even abusive to women. These attributes of the maternal healthcare system have remained fundamental to understandings of why many women continue to deliver their babies at home despite government efforts to ensure that all births take place in health facilities under the supervision of trained health professionals.

As access, especially equity of access and use of skilled maternal health interventions continues to be an essential ingredient to the reduction of maternal and newborn mortality and the attainment of the maternal and child health-related MDGs [8], there is an urgent need in Ghana to ensure that all pregnant and labouring women have full access to skilled care services as and when they need them. There is therefore the need for these services to be organised and delivered in a way that is medically appropriate, socially sensitive, and culturally responsive. This means that the extent to which the healthcare system in Ghana becomes more responsive to the pregnancy and birthing care needs of women including respect as reflected by the degree to which the health system is sensitive to women’s needs, dignity, confidentiality, choice and autonomy as well as the level of attention given to clients in terms of promptness, quality of environment, and access to social assistance, would be an important determinant of increased and equitable access to skilled maternal and newborn healthcare in Ghana.

## Consent

Written informed consent was obtained from the patient for the publication of this report and any accompanying images.
